# Claudin expression in the rat endolymphatic duct and sac - first insights into regulation of the paracellular barrier by vasopressin

**DOI:** 10.1038/srep45482

**Published:** 2017-04-04

**Authors:** Daniel Runggaldier, Lidia Garcia Pradas, Peter H. Neckel, Andreas F. Mack, Bernhard Hirt, Corinna Gleiser

**Affiliations:** 1 Institute of Clinical Anatomy and Cell Analysis, University of Tübingen, Tübingen, Germany

## Abstract

Hearing and balance functions of the inner ear rely on the homeostasis of the endolymphatic fluid. When disturbed, pathologic endolymphatic hydrops evolves as observed in Menière’s disease. The molecular basis of inner ear fluid regulation across the endolymphatic epithelium is largely unknown. In this study we identified the specific expression of the tight junction (TJ) molecules Claudin 3, 4, 6, 7, 8, 10, and 16 in epithelial preparations of the rat inner ear endolymphatic duct (ED) and endolymphatic sac (ES) by high-throughput qPCR and immunofluorescence confocal microscopy. Further we showed that Claudin 4 in the ES is a target of arginine-vasopressin (AVP), a hormone elevated in Menière’s disease. Moreover, our transmission-electron microscopy (TEM) analysis revealed that the TJs of the ED were shallow and shorter compared to the TJ of the ES indicating facilitation of a paracellular fluid transport across the ED epithelium. The significant differences in the subcellular localization of the barrier-forming protein Claudin 3 between the ED and ES epithelium further support the TEM observations. Our results indicate a high relevance of Claudin 3 and Claudin 4 as important paracellular barrier molecules in the ED and ES epithelium with potential involvement in the pathophysiology of Menière’s disease.

The endolymphatic sac (ES) is a non-sensory epithelial part of the inner ear that is connected with the cochlear and the vestibular organ through the endolymphatic duct (ED) (Fig. 1). The ES forms an extension of the endolymphatic fluid space. It is located in the petrous bone, partly extending into a meningeal duplicature in close proximity of the sigmoid sinus^1^. As opposed to the sensory part of the endolymphatic fluid space within the otic capsule that is surrounded by perilymph, the ED and ES are in direct contact with the extracellular fluid in the sigmoid sinus and the subarachnoid space. The ED and ES are thought to be involved in the regulation of the endolymphatic ion and fluid homeostasis^2^,^3^,^4^ for maintenance of hearing and balance functions, but the precise underlying mechanisms still remain to be elucidated.

In general, it is currently accepted that the ion and fluid homeostasis is regulated by a combination of transcellular and paracellular transport mechanisms through an epithelial barrier^5^,^6^. To date, a broad range of channels and transporters have been identified in the epithelium of the ES that might regulate the transcellular fluid and ion homeostasis, such as aquaporins^7^, sodium – potassium – chloride cotransporters (NKCC)^7^,^8^,^9^, the epithelium sodium channel (ENaC)^10^, pendrin (Slc26a4)^3^,^11^, the cystic fibrosis transductor regulator (CFTR)^12^, and K^+^ channels like KCNN2, KCNJ14, KCNK2, and KCNK6^13^. However, little is known regarding the mechanisms underlying the paracellular fluid and ion transport that is controlled by cell-cell contacts known as tight junctions (TJ) in the ED and ES. In tight junctions, the apical areas of adjacent cells are connected by a series of sealing strands composed of members of the claudin family and associated proteins such as ZO-1^14^,^15^,^16^, ZO-2^17^, ZO-3 and TAMPs (TJ-associated marvel proteins, occludin and tricellulin)^18^. As different claudins are involved either in increasing or decreasing the sealing strength of TJ, they can be considered as barrier^19^ or pore forming molecules^20^,^21^,^22^, respectively. Therefore, the combination of expressed claudins determines the final paracellular permeability property of the epithelial cell layer^23^. Pioneering work performed in 1981 using freeze fracture analysis or lanthanum injection experiments, showed that the ED and the proximal portion of the ES exhibit significantly fewer TJ strands and greater paracellular ion permeability compared to the distal portion of the ES^24^,^25^,^26^. Recently, a study visualized the inner ear fluid movements by light sheet fluorescence microscopy after the injection of fluorescein isothiocyanateedextran into scala media and found a remarkable uptake of the fluorescence marker into the endolymphatic duct and the periductal channels^27^. So far, only two studies investigated claudin mRNA expression levels in the ES epithelium by microarray analysis, RT-PCR analysis or *in*-*situ* hybridization^28^,^29^. However, almost no progress has been made in further elucidating the mechanisms of the paracellular fluid and ion homeostasis in the different parts of the ED or ES. In order to fully understand the underlying mechanisms, a more detailed understanding of the molecular structures of the epithelial barrier that separates the endolymph from the surrounding extracellular fluid space is essential. The first aim of this study was to analyze the expression of different members of the claudin family in the ED and to compare it with that of the ES to gain insights into the role of TJs in these epithelia.

Furthermore, several clinical and experimental studies suggest arginine vasopressin (AVP) as a potential regulator of endolymphatic fluid and ion homeostasis^30^,^31^,^32^. In collecting duct cells of the kidney AVP induces the translocation of vesicular aquaporin 2 (AQP2) into the luminal cell membranes via the vasopressin 2 receptor (V2R) and the cAMP/protein kinase A (PKA) pathway^33^. Since AQP2 and V2R expression have been shown for the ES, a similar mechanism has been proposed for endolymphatic fluid homeostasis and the development of Menière’s disease^7^,^34^. However, a direct functional characterization of such a mechanism is lacking.

Recent studies in other organs have linked the activation of the cAMP/PKA pathways to changes of the paracellular permeability and barrier properties^35^,^36^. Therefore, the second aim of our study was to analyze the effect of AVP on the claudin expression and subcellular localization in order to gain first insights into potential paracellular regulation mechanisms in epithelia of the ED and ES.

## Results

### Different ultrastructural morphology of TJs in the ED and ES epithelium

Ultrathin sections of ED and ES from young Wistar rats (postnatal day 4 (p4)) were subjected to transmission electron microscopy (TEM) analysis to investigate the ultrastructural morphology of TJ in the epithelium (Fig. 2). The TEM analysis of the ED revealed a mainly cuboidal epithelium with ribosome rich like cells connected with junctional complexes containing desmosomes and shallow TJ with only few kissing points (Fig. 2a). Some junctional complexes of the ED even appeared without kissing points (Fig. 2a’). Furthermore, the ribosome rich like cells showed basal invaginations and the ED epithelium was surrounded by a dense subepithelial capillary network (Fig. 2a) with endothelial cells connected by prominent TJ with numerous kissing points (data not shown). In contrast to the ED, the epithelium of the proximal sac portion and the intermediate sac portion of the ES contained two cell types, the ribosome rich and mitochondria rich cells. Between all of these cells TJs with numerous kissing points were observed (Fig. 2b and b’). We could only detect ribosome rich cells in the distal sac portion of the ES (Fig. 2c). Between the ribosome rich cells, TJ with multiple membranous kissing points were identified (Fig. 2c’). In contrast to the ribosome rich like cells in the ED, the epithelial cells of the ES regions did not show basal invaginations. However, as seen around the ED, subepithelial capillary network surrounded all regions of the ES network with endothelial cells connected by prominent TJ (data not shown). In summary, the TEM analysis revealed that the tight junctions of the ED epithelium were generally shorter and with considerably less membranous kissing points compared to the ES epithelium. Sporadically, some longer TJ with more kissing points were also identified in the observed ED preparations.

### Distinct mRNA expression of claudin genes in the ES and ED epithelium

Claudin mRNA expression was analyzed by high-throughput real-time TaqMan PCR analysis to identify the specific claudin genes forming the TJ and thus determining the paracellular permeability properties of the ED and ES epithelium. The surrounding tissue (blood vessels, meninges, connective tissue) adhering to the ES and ED preparations is exhibiting a significant claudin mRNA expression presumably due to capillary and meningeal TJ. Therefore, the ED and ES claudin mRNA expression was determined relative to the claudin mRNA expression of the surrounding tissue. The specific expression of the anion exchanger pendrin in the epithelium of the endolymphatic sac is well-studied and its expression clearly shown by different groups^11^,^37^,^38^ and hence served as a positive control target for our claudin mRNA expression analysis (Fig. 3). The epithelium of the ED and ES showed a stronger expression of Claudin 3, 4, 6, 7, 8, 10, 16, 17 and 22 compared to the surrounding tissue with normalized relative quantities (NRQ) of 10 or more (Fig. 3a). In contrast Claudin 5, 11 and 19 showed a stronger expression in the surrounding tissue compared to the epithelium of ED and ES. Most of the claudins identified in the ES epithelia were expressed by a factor of approximately two to five times stronger than in the ED, except for Claudin 17 and Claudin 22. Claudin 2, 14 and 18 mRNA expression could neither be found in the ED or ES nor in the surrounding tissue. To assess the absolute Claudin mRNA expression in ED, ES and surrounding tissue preparations of rat (p4) the qPCR cycle threshold (Ct) values were used (Ct values and the negative controls are visualized by a Heat Map in Supplementary Fig. S2). Targets that yielded Ct values between 14 and 17 in the high-throughput TaqMan qPCR screen were considered as moderately expressed. Claudins with Ct values >17 were ranked as weakly or very weakly expressed, whereas those with Ct values <14, such as the house keeping gene ubiquitin C (UBC), were considered to be expressed at high or very high levels (Table 1). For further investigations, only claudins were considered that fulfilled both of the following criteria: First, claudin mRNA expression had to be specifically expressed in ED or ES epithelia with a NRQ of 10 or more if compared with surrounding tissue. Second, the absolute expression estimated by the method described above had to be at moderate or higher levels. Claudin 3, 4, 6, 7, 8, 10 and 16 fulfilled both of the criteria and were therefore selected for further investigations. Very similar results were also obtained by analyzing the claudin transcriptome of adult rat (p31) ES specimens that are tightly attached to the surrounding meninges and therefore highly contaminated with surrounding tissue (Supplementary Fig. S1 and Supplementary Table S1).

Claudin 10 is known to exist in two major isoforms, resulting from two alternative exons, 1a and 1b (Claudin 10a, Claudin 10b)^21^,^39^. The two variants differ only in the first exon with Claudin 10b being two amino acids longer than Claudin 10a. In order to cover both Claudin 10 isoforms, a primer pair corresponding to the Claudin 10a sequence in exon 1 and a primer pair corresponding to the longer Claudin 10b sequence in exon 1 were generated for this study. Using these primer pairs in a RT-PCR analysis we showed that only the primer pair corresponding to Claudin 10b yielded a specific transcript (120 bp) in the epithelium of the rat ED and ES (Fig. 3b). The second primer pair however yielded no product. In kidney control samples both Claudin 10 mRNA isoforms were expressed.

### Differences in the expression of Claudin 4, 8 and 16 between the ED and ES epithelium

**Figure 1:**
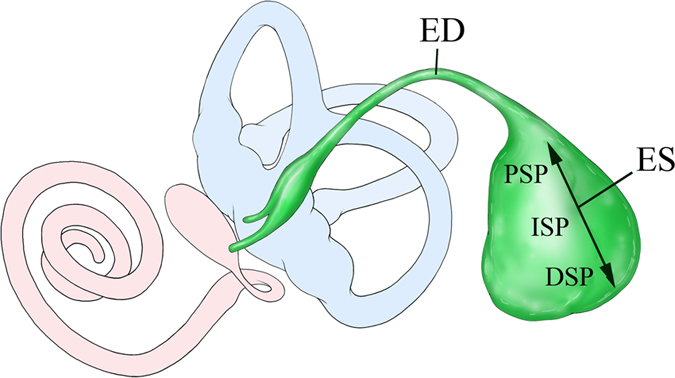
Schematic illustration of the membranous labyrinth of the inner ear represents the endolymphatic duct (ED; green) and sac (ES; green) and its relationship to the cochlea (red) and the vestibule (utricle and semicircular canals; blue). The magnified view shows the ES three parts, from anterior to posterior: the proximal sac portion (PSP), the intermediate sac portion (ISP) and the distal sac portion (DSP).

Immunofluorescence labeling of the selected claudins in whole mount ES preparations attached to the ED was performed to confirm the expression on protein level and to compare the claudin expression in the proximal and distal portions of ES (PSP and DSP, respectively; see Fig. 1) and ED. Claudin 3, 4, 6, 7, 8, 10 and 16 were expressed in the epithelium of ED and ES whereas the surrounding tissue did not yield any specific immunofluorescence labeling signal (Fig. 4). However, comparing the claudin expression between the ED and the parts of the ES (proximal sac portion and distal sac portion) epithelium revealed differences in the immunofluorescence intensity: Claudin 4 (Fig. 4b) and 16 (Fig. 4g) showed weaker fluorescence intensity in the ED whereas Claudin 4 (Fig. 4b’ and b”) and 16 (Fig. 4g’ and g”) immunoflourescence intensity was stronger in all regions of the ES. The immunofluorescence signal of Claudin 8 was strong between all cells in the ED (Fig. 4e), whereas in the parts of the ES only a few cells showed a scattered Claudin 8 labeling (Fig. 4e’ and e”). No differences between the ED and the different parts of the ES could be observed for the other selected claudins (Fig. 4a–a”). Mosaic images of optical sections covering the complete ES and adhering ED specimens stained with the particular claudin antibody acquired with structured illumination microscopy (apotome) confirmed these findings (Supplementary Fig. S2). Co-immunofluorescence labeling of the investigated claudins and ZO-1 on cryosections of the p4 rat ES and ED epithelium confirmed a differential expression of Claudin 4 and 16 between the ED and ES (Fig. 5). The co-immunofluorescence labeling of Claudin 4 and ZO-1 revealed a mainly cytoplasmic localization of Claudin 4 in the epithelia cells of the ES, with only a minor fraction co-localized with ZO-1 in the TJs (Fig. 5b–b”). In contrast, we could detect almost no expression of Claudin 4 in the epithelia cells of the ED (Fig. 5a–a”). Similarly, Claudin 16 exhibited a cytoplasmatic expression in the epithelia cells of the ES with no observable ZO-1 co-localization (Fig 5d–d”). In the epithelial cells of the ED, however, almost no Claudin 16 signal could be observed (Fig. 5c–c”). Claudin 8 labeling on cryosections of the ED and ES epithelium using different antigen-retrieval protocols and two different commercially available anti-Claudin 8 antibodies (Table 2) did not yield any positive Claudin 8 labeling (data not shown). Therefore, the data obtained from immunostaining of whole mount preparations for Claudin 8 (see Fig. 4e–e”) could not be confirmed on a subcellular level. In positive control tissue of the rat p4 kidney, an apical immunofluorescence labeling of Claudin 4 was observed in the thin ascending limb as reported in previous studies^40^,^41^,^42^. The expression of Claudin 16 in the positive control kidney was revealed in the cytoplasmic compartment of the thick ascending limb as previously reported^43^,^44^.

Consistent with previous reports^3^,^37^,^45^, immunolabeling of the anion exchanger pendrin served as a positive control that showed a polarized expression in the apical membranes of MRCs of the endolymphatic sac, but almost no expression in the cells of the ED (Supplementary Fig. S4). To determine whether Claudin 4 and 16 are also expressed in ES epithelium of mature rats (p31), co-immunolabeling of Claudin 4 or 16 together with ZO-1 was carried out on cryosections of p31 rat ES epithelium. Similar to the p4 ES a cytoplasmatic localization was detected in the adult ES epithelia cells (Supplementary Fig. S5).

### Differential subcellular expression of Claudin 3 in the ED and ES epithelium

Co-immunofluorescence labeling of the investigated claudins and ZO-1 on cryosections revealed differences in the subcellular localization of Claudin 3 between the ED and ES epithelium (Fig. 6): Claudin 3 was observed mainly in the basolateral membrane domains of the ED epithelium with only a minor fraction co-localized with ZO-1 (Fig. 6a–a”). In contrast, in the ES epithelium Claudin 3 co-localized mainly with ZO-1 and only a minor fraction distributed over the non-junctional cell membrane (Fig. 6b(b-b”)). Furthermore, the immunofluorescence analysis of mature p31 ES epithelium showed a strict co-localization of Claudin 3 with ZO-1 (Supplementary Fig. S5), which corresponds with the data obtained from the rat p4 ES specimen (Fig. 6). In the positive control tissue, co-immunoflourescene labeling of Claudin 3 with ZO-1 was revealed in the medullary thick ascending limb of the kidney as reported previously^40^.

### Subcellular localization of Claudin 6, 7, and 10 in the ED and ES epithelium

The analysis of co-localization of Claudin 6, 7, or 10 with ZO-1 on cryosections of the p4 rat ED and ES epithelium showed no differences in the expression or subcellular localization between the ED and ES epithelium (Fig. 7). Claudin 6 was expressed in the cytoplasma of the epithelial cells of the ED and ES (Fig. 7a–a”’and b–b”’). Claudin 7 was detected in the basolateral membranes of the epithelial cells in the ED and ES, and only a faint co-localization labeling of Claudin 7 and ZO-1 in a few cells of both epithelia could be observed (Fig. 7c–c”’and d–d”’). Claudin 10 was co-localized with ZO-1 in the epithelial cells of the ED and ES. Additionally, a strong cytoplasmic Claudin 10 immunofluorescent signal was detected in all of these cells (Fig. 7e–e”’ and f–f”’).

To determine whether Claudin 6, 7, and 10 are also expressed in ES epithelium of functional mature rats (p31), co-immunolabeling of Claudin 6, 7 or 10 proteins and ZO-1 was carried out on cryosections of p31 rat ES epithelium. Similar results regarding the Claudin 6, 7 and 10 protein expression and subcellular localization were obtained as compared to the p4 ES samples (Supplementary Fig. S5).

The confocal microscopic analysis of the subcellular localization of Claudin 6, 7 and 10 in rat p4 kidney control tissue, revealed similar results as were observed in rat p4 and p31 ED and ES and matched with other Claudin expression studies in the kidney^21^,^43^,^46^,^47^. However, in contrast to the adult ES, none of the former studies visualized Claudin 6 by immunostaining in any nephron segment in rat adult kidney^47^,^48^.

### Synthetic arginine-vasopressin (AVP) analogon 1-Desamino-8-D-Arginin-Vasopressin (dDAVP) induces the translocation of Claudin 4 in the TJs of the ES epithelia cells

Recent studies showed that cAMP/AMP regulates cytoplasmic expressed claudins in various tissues^35^,^49^,^50^. Further, it is known that AVP regulates the volume homeostasis of the ES via the cAMP/PKA pathway^8^,^30^,^51^,^52^, but a specific protein target regulating the paracellular water and ion homeostasis has not yet been identified. Therefore, analysis of the cAMP regulated claudins 4 and 16 by immunofluorescence microscopy was performed in organotypic ES cultures treated with the V2R agonist dDAVP or the V2R-antagonist H9400 as a control. When treated with 10^−6^ M H9400 for 3 h, Claudin 4 showed a clear cytoplasmatic distribution in the ES cells (Fig. 8a–a”’). However, in ES specimens treated with 10^−6^ M dDAVP for 3 h, the Claudin 4 and ZO-1 co-labeling WO: increased markedly and the basolateral membranes relative to the cytoplasmic Claudin 4 labeling (Fig. 8b–b”’). The stimulation of ES with lower concentrations of dDAVP (10^−8^ M and 10^−10^ M) and shorter time-periods (30 min or 1 h) did not yield a clearly observable translocation of Claudin 4 into the basolateral membranes of the epithelial cells. The exposure of the ES with dDAVP or H-9400 did not show any effect on the Claudin 16 labeling which was observed under all conditions in the cytoplasmatic compartment (data not shown).

## Discussion

**Figure 2:**
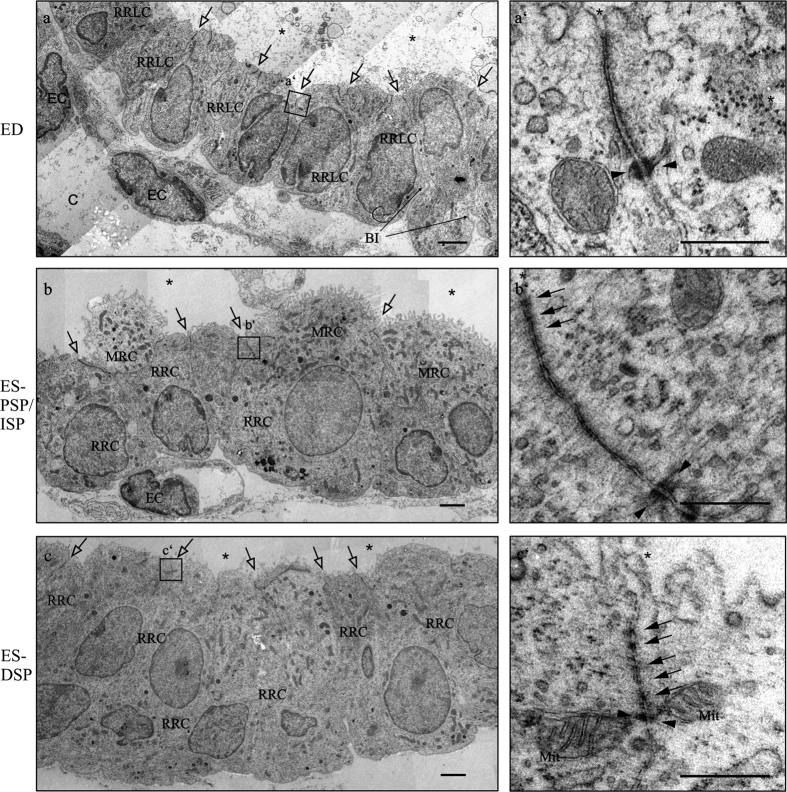
Transmission electron microscopy of the endolymphatic epithelia of rat p4 ED, PSP/ISP and DSP. (**a**) ED epithelium displaying cuboidal ribosome rich like cells (RRLC) connected with junctional complexes (black arrows) and capillaries (**c**) beneath the epithelium. The ED epithelium basal infoldings (BI) are clearly visible. a’) High magnification image displaying a junctional complex of the ED containing a desmosome (black arrow heads) but no membraneous kissing points. (**b**) Proximal sac portion (PSP) and intermediate sac portion (ISP) epithelium containing mitochondria rich cells (MRCs) and ribosome rich cells (RRCs) all connected with junctional complexes. (b’) High magnification image showing a long junctional complex with several membraneous kissing points (empty arrows) and 2 desmosomes. (**c**) Distal sac portion (DSP) epithelium containing ribosome rich cells (RRCs) connected with junctional complexes. (c’) High magnification image showing a junctional complex between two ribosome rich cells (RRCs) containing at least five kissing points and a desmosome in close proximity. *BI* basal invagination; *EC* endothelial cell; *Mit* mitochondrion. Scale bars A–C: 2 μm; A’–C’ 500 nm.

In recent decades a key role for the endolymphatic sac in the maintenance and regulation of the endolymphatic ion and fluid homeostasis within the inner ear has been identified^3^,^53^,^54^,^55^. In most recent studies, the research has mainly focused on the transcellular mechanisms mediating the ion and fluid transport of the inner ear^8^,^38^,^56^,^57^. However, little focus was laid on elucidating the paracellular mechanisms underlying the endolymphatic fluid and ion homeostasis as well as identifying the role of the various parts of the ED and ES in these processes. Pioneering freeze-fracture analysis performed in the 1980s suggests the ED and PSP of the ES as the location of endolymph resorption based on TJ complexes that were morphologically described as sparsely developed and shallow compared to the distal areas of the ES^24^,^58^. In our study we confirm the presence of shallow TJ in the ED by conventional TEM analysis, whereas more complex TJ with numerous membranous kissing points were found in the distal ES epithelium (Fig. 2). Moreover, using an extended qPCR analysis as well as immunohistochemical analysis, we found clear differences in the expression of several claudins between ES and ED.

**Figure 3:**
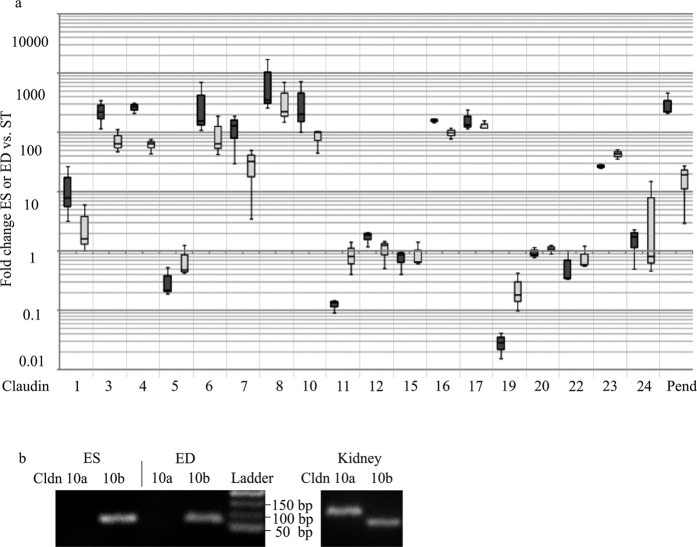
Claudin mRNA expression in rat p4 ED and ES epithelium. (**a**) High throughput qPCR analysis of ED and ES mRNA. TaqMan probes against exon-exon boundaries of all mRNA subtypes were used (Table 1). Box-whisker-blot shows 25–75 percentile of the normalized relative quantities (NRQs) of rat p4 ED (n = 3; light gray bars) or ES (n = 3; black bars) claudin mRNA expression versus claudin mRNA expression of surrounding tissue (ST). Claudin 3, 4, 6, 7, 8, 10, 16, 17 and 23 mRNA were found to be expressed considerable stronger in the ED and ES with a NRQ of 10 or more if compared with surrounding tissue (ST). Claudins not shown were not expressed in the ES or ED (n = 3). Positive control target: Pendrin (Pen). (**b**) Cropped gel images of RT-PCR analysis of the claudin 10a and 10b mRNA isoforms for rat p4 ED and ES epithelium. Only Claudin 10a transcripts were obtained, whereas the Claudin 10b primers yielded no transcript in ED and ES. Both transcripts, Claudin 10a and 10b, were obtained in the positive control tissue of rat p4 kidney. DNA Ladder: 50 bp.

**Figure 4:**
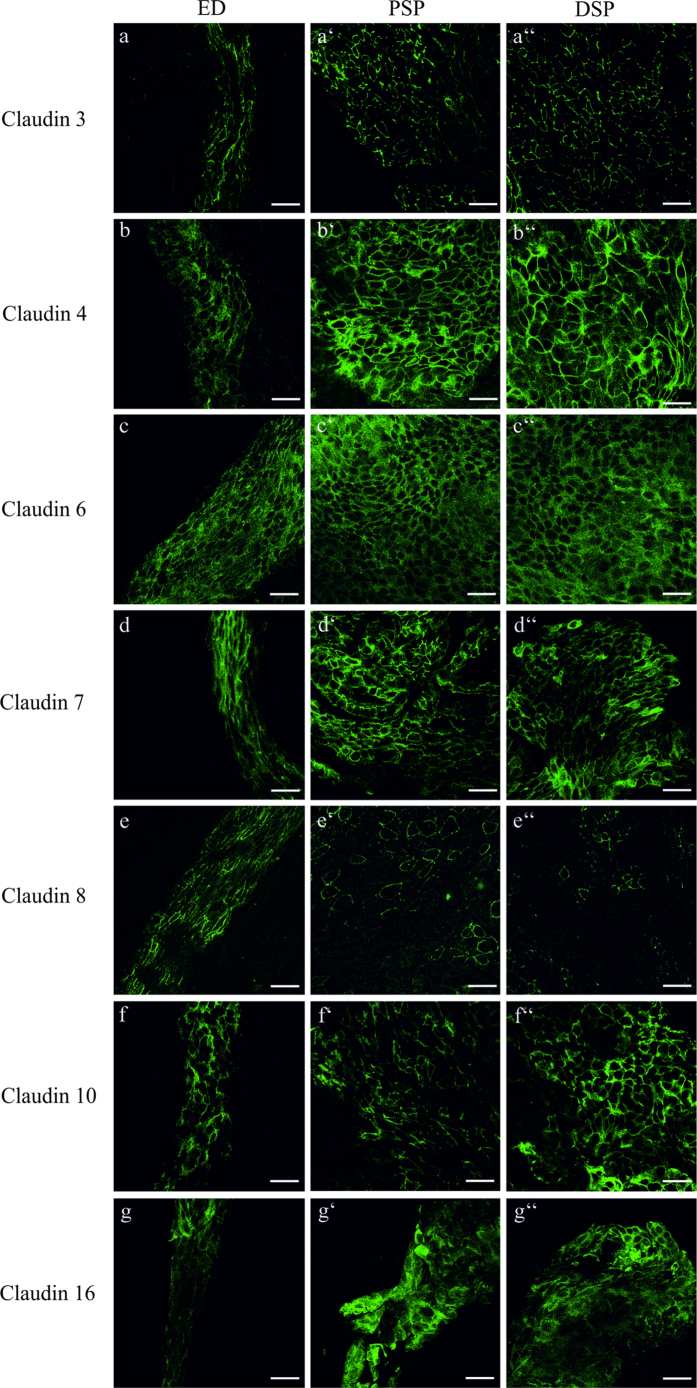
Cellular expression of claudin proteins in the ED and ES epithelium of p4 rat. Whole mount preparations from rat p4 ES with adjacent ED were stained with the indicated claudin subtype antibody followed by Alexa 488-conjungated anti-rabbit antiserum. (**a**–a”) Claudin 3 was expressed in the membranes of the epithelia cells of the ED and the PSP and DSP of the ES, whereas no differences in the Claudin 3 signal intensity between the different ES portion and the ED could be observed. (**b**–b”) Claudin 4 and (**g**–g”) Claudin 16 immunofluorescence signal was weaker in the cytoplasm and membrane of the epithelial cells of the ED than in the cells of the PSP and DSP. (**c**–c”) Claudin 6 staining yielded a strong cytoplasmic immunofluorescence signal in the epithelial cells of the ED and ES portions. (**d**–d”) Claudin 7 immunolabeling was observed in the membranes and in a lower intensity in the cytoplasma of the ED and ES epithelium. (**e**–e’) Claudin 8 expression was strong in the membranes of the epithelial cells of the ED whereas in the PSP only a few cells showed a Claudin 8 membrane expression. (e”) In the DSP a membrane expression of Claudin 8 was only observed in very few cells. (**f**–f”) Claudin 10 was expressed in the membranes of the ED and ES epithelium. Scale bars: 10 μm.

**Figure 5:**
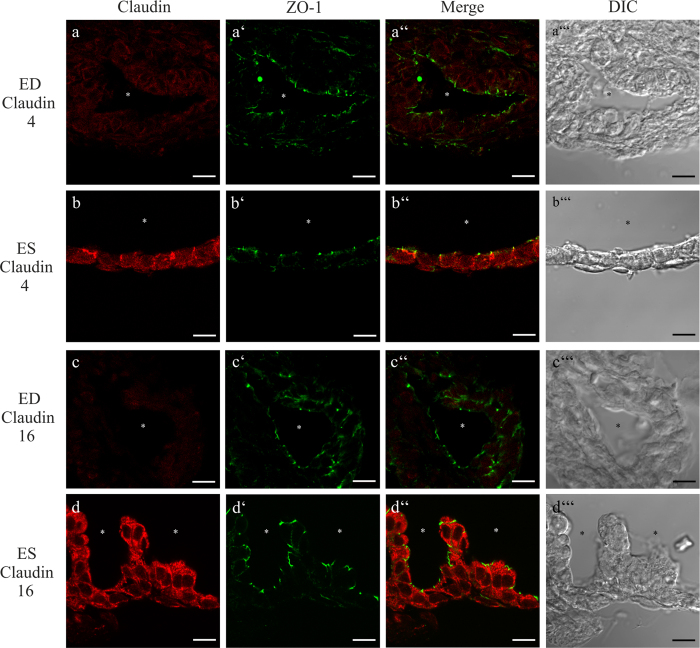
cLSM analysis of the subcellular immunolocalization of Claudin 4 and 6 in rat p4 epithelial cells of ED and ES. Cryosections of rat p4 ED and ES were co-localized with the indicated claudin subtype antibody and ZO-1 antibody. (**a**–a”’) Very low cytoplasmic Claudin 4 expression in the epithelial cells of ED. No co-localization with ZO-1 could be observed. (**b**–b”’) Cytoplasmic Claudin 4 expression was found in the ES epithelium. In a few cells Claudin 4 co-localized with ZO-1. (**c**–c”’) The epithelium of the ED lacked Claudin 16 labeling. (**d**–d”) The epithelial cells of the ES exhibited a cytoplasmic Claudin 16 immunolabeling with a minor fraction co-localized with ZO-1. Scale bars: 10 μm.

The analysis of the subcellular distribution of Claudin 3 provides an explanation for the ultrastructural differences of TJ in the ED and ES observed by TEM analysis (Fig. 2): Claudin 3, a molecule expressed in a wide variety of different epithelia^40^,^59^, was found to be an essential component in the blood-brain^60^ as well as the blood-testis barrier^61^. In that context, Claudin 3 was characterized as a strong paracellular barrier molecule for charged and uncharged molecules and its overexpression resulted in a significant increase in number and complexity of TJ sealing strands^19^. In our study Claudin 3 in particular co-localized with ZO-1 in TJ of the ES epithelium whereas in the ED it was primarily found in the basolateral membrane domains. A localization of Claudin 3 as a non-TJ Claudin has already been described in other organs such as the rat intestine^59^ or the human endometrium^62^. In the endometrium a complex network of TJ strands^63^, high transepithelial resistance and localization of Claudin 3 in the TJ were observed in the proliferative phase of the female cycle^62^. In contrast, in the secretory phase the TJ were characterized by a disordered network of strands with reduced depth^63^, decreased transepithelial resistance and a distribution of Claudin 3 into the basolateral membrane domains^62^. Hence, the basolateral localization of Claudin 3 could possibly account for the reduced number and complexity of TJ sealing strands seen in the ED region^24^,^26^. In contrast, the localization of Claudin 3 in the TJ of the ES could explain the complex TJs with numerous membranous kissing points observed in the distal sac portion in our and former studies by electron microscopic analysis^24^,^64^. As for the other claudins, we did not find any striking differences in the subcellular localization, but rather in the quantity of mRNA and protein expression. Claudin 3, 4, 6, 7, 8, 10 and 16 mRNAs were expressed by a factor of 2–5 less in the ED compared to the ES (Fig. 3) and in the confocal immunofluorescence microscopy we found lower signal intensity for Claudin 4 and 16 in the ED compared to the ES region (Fig. 5). The lower expression of claudin molecules together with the basolateral expression of Claudin 3 (see above) could be an explanation for the reduced number of TJ sealing strands observed in the ED region. Based on these results, it is not only possible that the ED epithelium exhibits lower paracellular barrier properties compared to the ES but it might also indicate that the endolymphatic fluid and ion homeostasis may be regulated in the ED rather than in the ES. This hypothesis is supported by several other studies: experiments with fluid tracer dyes demonstrated a lower paracellular barrier in the ED epithelium compared to the ES region^25^,^65^. A more recent study demonstrated a remarkable paracellular uptake of fluorescein isothiocyanateedextran injected to the scala media into the endolymphatic duct and the periductal channels^27^. Morphological examination of the endolymphatic duct and the surrounding periductile channels from archival human temporal bones^66^ and surgical specimens^67^ shows that these structures form a network and suggests that this ED tissue network may play a primary role in endolymph absorption^66^,^67^. Measurements of the ion compositions showed high potassium and low sodium concentrations in the cochlear endolymph, whereas the opposite was reported in the case of the endolymph of the ES^68^,^69^,^70^,^71^. It is possible that the endolymph equilibration, especially regarding small molecules and ions, already occurs in the ED, by paracellular mechanisms.

Apart from studies examining the ultrastructural morphology of TJ in the ED and ES, few studies were performed to elucidate the claudin mRNA expression as the molecular determinants of the TJ. In our study, a high-throuput qPCR screen was used to identify the TJ molecules expressed in the ED and ES epithelium. We found a strong and specific mRNA expression for Claudin 3, 4, 6, 7, 8, 10 and 16 (Fig. 3). These data are in line with a microarray study that could also identify Claudin 3, 4, 8, 10 and 16 mRNA expression in the adult rat ES tissue^28^. It is important to note that we also detected a low expression of several other claudins in the analyzed endolymphatic epithelia such as Claudin 1, 12, 15, 17, 20, 23 or 24. However, in the selection process those claudins did not fulfill the required criteria and were dropped from the list, but we cannot exclude a role of those claudins in the paracellular barrier of the ED and ES epithelia. As for Claudin 2, 14 and 18 we could not find any expression in the analyzed tissues in contrast to another study^29^.

**Figure 6:**
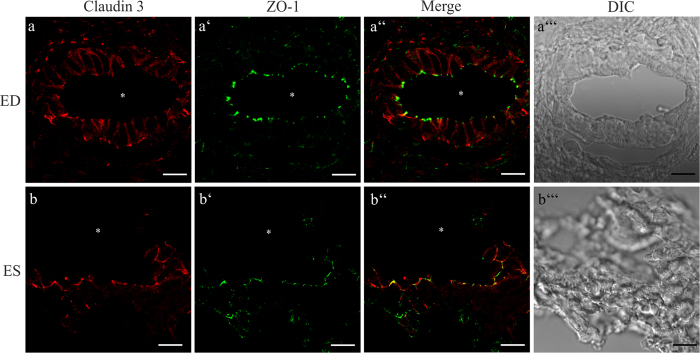
cLSM analysis of Claudin 3 labeling in cryo-sections of the rat p4 ED and ES epithelium. Cryosections of rat p4 ED and ES were co-localized with the indicated claudin subtype antibody and ZO-1 antibody. (**a**–a”’) Epithelial cells of the ED exhibit a mainly basolateral expression of Claudin 3. (**b**–b”’) while in the ES epithelium Claudin 3 is co-localized with ZO-1 in the TJs. Scale bars: 10 μm.

**Figure 7:**
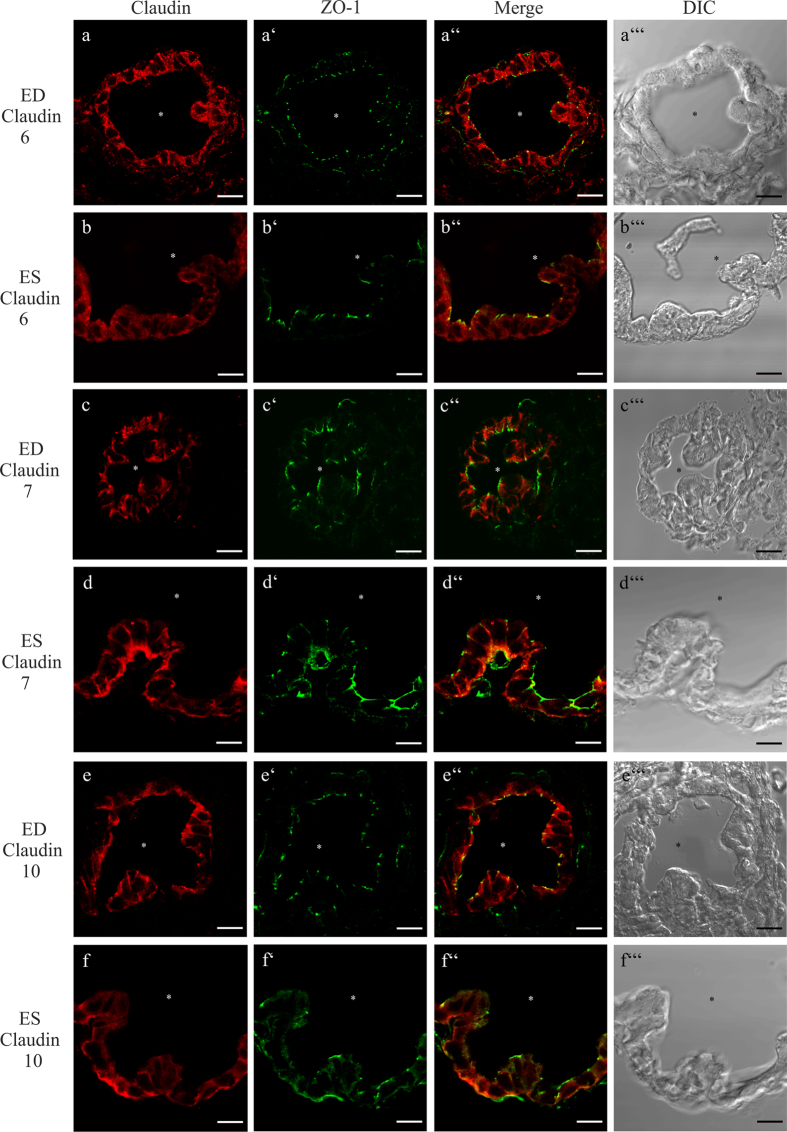
cLSM analysis of the subcellular immunolocalization of Claudin 6, 7 and 10 in the epithelial cells of p4 rat. Cryosections of rat p4 ED and ES were co-localized with the indicated claudin subtype antibody and ZO-1 antibody. (**a**–a”’ and **b**–b”’) Claudin 6 immunolabeling was detected in the cytoplasm of the ED and ES epithelial cells. (**c**–c”’ and **d**–d”’) Claudin 7 immunolabeling was localized in the basolateral membranes of the ED and ES epithelial cells. Additionally, a faint Claudin 7 expression was found within these cells. (**e**–e”’ and **f–**f”’) Claudin 10 was co-localized with ZO-1 in the epithelial cells of ED and ES, the cells of both epithelia exhibited additional cytoplasmic immunolabeling. Scale bar: 10 μm.

**Figure 8:**
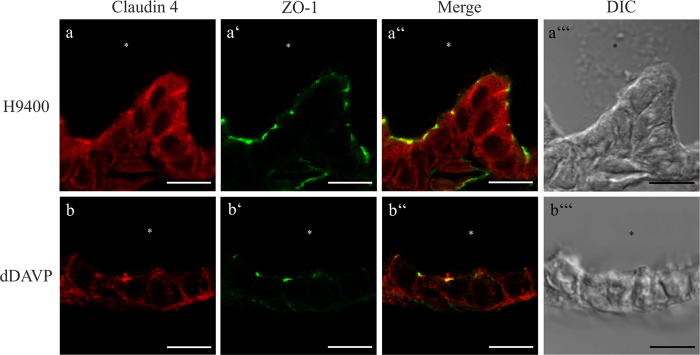
Immunolabeling of dDAVP induces the translocation of Claudin 4 into the TJs of the ES epithelia cells. Confocal LSM analysis of the subcellular translocation of Claudin 4 in cryosections of the rat p4 ES epithelium. (**a**–a”’) After treatment with the V2R antagonist H9400 Claudin 4 labeling was observed in the cytoplasm of ES epithelial cells. (**b**–b”’) After dDAVP treatment, intracellular labeling was markedly reduced, whereas co-localization with ZO-1 in the TJs and labeling of the basolateral membranes has increased. Scale bar 5 μm.

By analyzing the protein expression of the selected claudins with confocal immunofluorescence microscopy we could confirm the qPCR data and demonstrate a highly specific protein expression in the analyzed endolymphatic epithelia (Figs 5,6 and 7). Claudin 10 was co-localized with ZO-1 at the apical poles of the lateral membranes and the cytoplasm of the epithelial cells of ED and ES. It is known that Claudin 10 exists in two major isoforms 10a and 10b with different paracellular barrier properties^21^. In the ED and ES epithelia Claudin 10b was expressed, a molecule shown to significantly increase the paracellular cation - especially Na^+^ - permeability^21^,^39^. The maintenance of the endolymphatic Na^+^-homeostasis in the inner ear is essential since the transduction channels of the hair cells are cation-permeable, but not K^+^-selective^72^. Dysfunction of the Na^+^ transport in the inner ear was found to cause sensory hair cell dysfunction or endolymphatic hydrops^73^. Most Na^+^ flow studies in the epithelial cells of the ES have focused on transcellular transport mechanisms^3^,^74^,^75^. The observed expression of Claudin 10b in the ES epithelium suggests a possibility for the passive paracellular diffusion of Na^+^. Claudin 10a, which has been suggested to increase the anion permeability^21^,^39^, could not be detected in the analyzed endolymphatic epithelia. This is confirmed by other studies demonstrating a restricted expression of Claudin 10a to the cortical region of the kidney and the uterus^21^,^39^. Claudin 7 was expressed mainly on basolateral membrane domains of the ED and ES epithelia cells, rather than co-localized with ZO-1 in the TJs. This is in agreement with other reports investigating its subcellular localization such as in mouse intestinal epithelium^76^, human duodenum and jejunum^77^, or mouse kidney^78^. The function of this non-TJ claudin is not well understood but as an extrajunctional claudin it might serve as a storage pool for junctional claudins^76^, contribute to cell-cell signaling^79^, or mediate cell-matrix interactions^80^. Interestingly, in our study several of the identified claudins, namely Claudin 4, 6, and 16, were localized mainly in the cytoplasm rather than in membranes or TJ of the endolymphatic epithelium. The translocation of cytoplasmatic claudins into TJ and vice versa due to posttranslational modifications has already been described in several reports as a mechanism to regulate the paracellular barrier and permeability properties^49^,^81^,^82^,^83^. Therefore, we investigated if those principles also applied to the endolymphatic epithelia of the ES. Claudin 4, a protein expressed in a variety of epithelial tissues^40^,^59^,^84^,^85^,^86^, was found to act primarily as a paracellular barrier molecule with overexpression resulting in an increased complexity and number of TJ sealing strands^84^,^87^. We showed that the translocation of Claudin 4 from the cytoplasm into the basolateral membranes and TJ of the epithelia cells was induced in ES specimens stimulated with the AVP analogon dDAVP. In a variety of studies abnormally high plasma levels of AVP were correlated with Menière’s disease^32^,^51^,^88^,^89^. Menière’s disease is a common clinical syndrome of recurrent attacks of vertigo, fluctuating hearing loss and tinnitus^90^,^91^. The pathologic hallmark of Menière’s disease is a volume distention of the endolymphatic fluid space designated as endolymphatic hydrops EH^90^,^91^,^92^. The pathophysiological basis for the disturbed endolymph homeostasis in endolymphatic hydrops remains controversial^93^. Causal treatment options for endolymphatic hydrops are not available. However, it remains arguable whether the observed high AVP concentration associated with endolymphatic hydrops is causative or an epiphenomenon^94^,^95^. The transcellular route similar to the V2R-mediated AQP2-translocation mechanism in renal physiology^33^ has been proposed so far as a mechanism for the endolymphatic fluid and ion homeostasis and the development of an endolymphatic hydrops^51^,^96^. However, a most recently published study showed that AVP dynamically increases the paracellular permeability in the thick ascending limb of Henle, suggesting claudin proteins as the molecular mechanism of this regulatory effect^43^. In our study we could demonstrate a role of dDAVP in the regulation of the TJ molecule Claudin 4. This result indicates that fluid and ion homeostasis of the endolymphe is fine-tuned by AVP regulating the paracellular barrier of the ES epithelium. Several findings support the theory that the ES endolymphatic water homeostasis is regulated via a V2R-mediated mechanism. Radiotracer studies demonstrate AVP binding to the human ES epithelium^97^,^98^. Rat^99^,^100^ and human ES epithelia express V2-R and AQP2 mRNA and protein^34^,^101^. Experimental EH can be induced by systemic application of AVP in adult guinea-pigs *in vivo*^102^,^103^,^104^ and reduced by V2-R antagonists^102^,^105^ or lithium^106^. These findings led to the hypothesis that patients with Menière’s disease respond more sensitively to fluctuations in the AVP plasma concentration^107^. Based on our results we therefore suggest, a role for the AVP-mediated regulation of the paracellular epithelial barrier in the ES, in the endolymphatic ion and fluid homeostasis. Mal-regulation of this mechanism may result in an endolymphatic hydrops. dDAVP was shown to specifically bind and activate the V2R by inducing the production of cAMP with the subsequent activation of the PKA^33^.

Claudin 16, a molecule expressed only in a limited number of tissues^108^,^109^ and suggested to be involved in the paracellular transport of bivalent cations^110^, was reported to be regulated via the same PKA pathway^49^. Interestingly, a translocation of Claudin 16 after dDAVP stimulation of ES specimens was not found (data not shown). Based on these results in the ES epithelium, dDAVP might regulate the Claudin 4 translocation via a different pathway than by the PKA/cAMP pathway. A recent study demonstrated the regulation of a variety of sodium transporters by AVP via the V2R/WNK4/SPAK and OSR pathway^111^. Other data also showed that activation of the kinase WNK4 induced the phosphorylation of different claudins such as Claudin 1, 3, 4 and 7 and thereby modulated the paracellular permeability properties of different epithelial cells^82^,^83^. Therefore it seems reasonable to assume that dDAVP might stimulate the V2R/WNK4 pathway rather than via cAMP/PKA to induce the translocation of Claudin 4. However, further studies investigating the regulating effects of these pathways on Claudin 4 are necessary.

In a high throughput qPCR screen, adult ES specimen containing adhering surrounding tissue was compared to surrounding tissue alone to identify claudins that are specifically expressed in adult ES epithelia (Supplement Fig. 1 and Table 1). Adult ES epithelia form a tubular network rather than a sac like structure and tend to adhere tightly to surrounding tissue^112^, which makes it difficult to obtain adult ES preparations with minimal contamination and it is not possible to precisely separate the ED from the adult ES. The results of this qPCR analysis showed that the obtained data from p4 ES epithelium is comparable to the claudin expression in adult ES tissue. Moreover the analysis of tissue sections of p31 ES specimen stained for the selected claudins by confocal immunfluorescence microscopy (Supplement Fig. S5) yielded comparable claudin expressions in the adult ES specimen and the ES p4 samples. These similarities between ES p4 and ES p31 samples indicate that the Claudin 4 translocation seen in dDAVP-treated p4 organotypic ES cultures might also apply for adult endolymphatic epithelia.

In conclusion, using TEM-analysis we found that the epithelial cells of the ED are connected by “leaky” tight junctions. A possible explanation for this “leaky” ED epithelium is the lower expression of claudin mRNA and the distribution of the strong barrier protein Claudin 3 in the basolateral membrane domains instead of the TJs in ED epithelial cells. Therefore, we suggest that the ED epithelium facilitates paracellular pathways and plays a primary role in the regulation of the endolymphatic fluid and ion homeostasis. Further we showed that the dDAVP/V2R- mediated translocation of cytosolic Claudin 4 into TJs is a mechanism capable to regulate the paracellular barrier and permeability properties of the ES epithelium associated with Menière’s disease.

## Material and Methods

### Animals and tissue preparation

Wistar rats obtained from Charles River (Sulzfeld, Germany) were maintained in an in-house animal facility. Animals were handled and all methods were carried out in accordance to the institutional guidelines of the University of Tübingen, which conform to the international guidelines. All experimental protocols were approved by the Committee for Animal Experiments of the Regional Council (Regierungspräsidium Tübingen). The animals (either at postnatal day 04 or postnatal day 31) were suffocated with carbon dioxide (CO_2_) and decapitated. The temporal bones were removed immediately, the skull divided in the midline and placed in ice-cold phosphate bufferd saline without Ca^2+^ and Mg^2+^ (PBS; Sigma Aldrich, Munich, Germany). Next, the brain was removed in order to access the temporal bone and the posterior fossa. The entire ES including the ED was removed from the temporal bone. Care was taken to obtain a pure epithelial preparation without any surrounding tissue adhering to the ES. For high throughput RT-PCR, the ES was separated from its duct with fine scissors in ice cold PBS without Ca^2+^ and Mg^2+^ (Sigma Aldrich). Surrounding tissue in close proximity to the ES containing meninges, connective tissue and blood vessels was used as control tissue. For immunohistochemical analysis and ultrastructural analysis the ES and adhering ED were kept intact and were fixed in the appropriate fixation solution. Positive control tissue from the kidney was obtained from the same rats from which the ED and ES were removed.

For pharmacological stimulation, the ES specimens were incubated immediately after preparation in DMEM/F12 medium without HEPES supplemented with 25% horse serum, 10 IU/ml penicillin/streptomycin, 2 mM L-glutamine, 0,9 mg/ml sodium bicarbonate (all ThermoFisher Scientific) and the appropriate pharmacon.

### Transmission electron microscopy (TEM)

For ultrastructural analysis of rat (postnatal day 4–p4) ED and ES, ES preparations including the ED were fixed in 2.5% glutaraldehyde in 0.1 M cacodylate buffer (pH 7.4) containing 0.1 M sucrose for 30 min at 4 °C. Next, the specimen were washed three times with 0.125 M cacodylate buffer for 30 min. Afterwards, preparations were fixed with 1% osmium tetroxide (OsO_4_) in 0.1 M cacodylate buffer for 1 h at room temperature, followed by dehydration in ethanol (50–100%). Next, the samples were transferred to propylen oxide (10 min propylen oxide (1:3), 30 min Epon propylen oxide (1:3), 30 min Epon propylen oxide (1:1), 60 min Epon propylen oxide (3:1) and finally 90 min in Epon (Epoxy Embedding Medium Kit; Sigma-Aldrich). The polymerization reaction was performed for 3 days at 55 °C. Ultrathin sections were counterstained with ethanolic uranyl acetate and lead citrate, and analyzed in a transmission electron microscope (LEO EM912 Omega electron microscope; Zeiss, Oberkochen, Germany). Images were obtained with a slow-scan CCD camera (PROSCAN, Germany; analySIS pro imaging software, version 3.2) and processed with Adobe Photoshop CS (Adobe Systems Software, Dublin, Ireland). For analysis of the TJ morphology 30 TJ of each part of the ED and ES were evaluated.

### RT-PCR and high throughput real-time PCR of ED and ES preparations

RT-PCR was used to detect the Claudin 10a and 10b isoform expressed in the ED and ES of rats (p4). The total RNA of surrounding tissue pooled from 12 specimens, 12 pooled ED and 12 pooled ES samples were isolated by disrupting the tissues using a minilysis system (VWR Life Science Competence Center, Erlangen, Germany) and the precellys tissue RNA kit (VWR Life Science Competence Center). RNA was treated with with DNase I peqGOLD (VWR Life Science Competence Center). RT-PCR was performed with the cDNA synthesis Kit H Plus (VWR Life Science Competence Center) and TaqDNA Polymerase Kit (VWR Life Science Competence Center). Primers used were as follows: Cldn10a: forward 5′-TTTGTGGGAGTCCTGTCCAG-3′ and reverse 5′-CCACACACCAGAGCTGAGAT-3′; Cldn10b: forward 5′-AGGACTTCCCCTCCATGCT-3′ and reverse 5′- ACCGCGGCAATCATTAGTC-3′. For negative controls, the RNA solution was replaced with nuclease-free water. Samples were denatured at 94 °C for 2 minutes, followed by 35 PCR cycles at 94 °C for 30 seconds, annealing at 60 °C for 30 seconds, strand synthesis at 72 °C for 45 seconds, and final extension at 72 °C for 7 minutes. The PCR products were analysed with standard electrophoresis on 1,75% agarose gels at 110 V, stained with PeqGreen (VWR Life Science Competence Center). The size of each PCR product was estimated by using a PeqGold 50 bp DNA Ladder (VWR Life Science Competence Center) In order to analyze the claudin transcriptome of the ED- and ES epithelium, a reverse transcription and preamplification reaction was performed by the Cells Direct Kit (Thermo Fisher scientific, St. Leon-Roth, Germany). The primers/probes used to quantify mRNA expressions of claudin genes were acquired from TaqMan^®^GenExpression assays (Thermo Fisher scientific) as summarized in Table 2. 20 ng of isolated RNA obtained from ED, ES or ST samples were transferred into 9 uL of preamplification reaction mix (5 μL 2x RXN direct buffer, 1.3 μL TE buffer pH 7, 0.2 μL Superscript III, 2.5 μL of 1:100 diluted 20x assay mixes to achieve a final volume of 10 μL and reverse transcription performed for 15 min at 50 °C (VWR Doppio Thermal Cycler; VWR International, Darmstadt, Germany). Next, the obtained cDNA was preamplified with 18 PCR cycles each with 15 sec at 95 °C and 4 min at 60 °C. After preamplification, samples were diluted 1:5 in TE-buffer and 2.7 μL added to 3.3 μL of sample mix (3 μL of universal PCR master mix with no emperase (Thermo Fisher scientific) and 0.3 μL of GE sample mix (Thermo Fisher scientific). 20x assay mixes were diluted 1:2 with 2x assay reagent (Thermo Fisher scientific). For negative controls, the reverse transcriptase (Superscript III) was replaced with nuclease-free water. Next the preamplified samples and diluted assays were loaded on a 48.48 Dynamic Array™ (Fluidigm, München, Germany) and qPCR performed by the Biomark HD system (Fluidigm). Measurements were conducted in triplicates, and a no-template blank served as the negative control. The data were collected with BioMark HD Data Collection software (Fluidigm) and Ct values were exported to Excel.

### qPCR data evaluation and statistical analysis

RT-qPCR Ct values were used to calculate the normalized relative quantities (NRQs) according to the modified ΔΔCt method^113^ using UBC and TBP as reference genes. The expression levels of the mRNAs from three independent experiments each using the pooled RNA of 12 ES and 12 ED samples were reported as fold changes vs. control (ST). These normalized relative quantities (NRQs) were used to create a box – whisker - blot with the calculated 25^th^ and 75^th^ percentile.

### Immunofluorescence microscopy

Whole-mount preparations of the ES including the ED from rats (p4) for claudin proteins and ZO-1 labeling were fixed in a 4% paraformaldehyde solution (Carl Roth, Karlsruhe, Germany) for 30 min. The whole-mount preparations were incubated with the appropriate primary antibody (1:100; Table 3) in 0.1% NDS in PBS. Antibody binding was visualized with AlexaFluor™ 488- or AlexaFluor™ 546- conjugated antibodies (1:400; Table 3) in the same dilution buffer used for primary antibodies. Next, ES specimen were mounted on SuperFrost^®^plus glass slides (Langenbrinck, Emmendingen, Germany) and embedded with FluorSave™ reagent (Merck Millipore, Darmstadt, Germany).

For immunofluorescence analysis of Claudin proteins in rat ED (p4), ES (p4 and p31) and kidney cryo-sections, specimens were fixed in a 4% paraformaldehyde solution (Carl Roth) for 30 min. Following cryoprotection in 30% sucrose in PBS, the specimen were embedded in a cryo-gel (Tissue-Tec O.C.T. compound; Sakura Finetek, Zoeterwoude, Netherlands). The frozen sections (12 μm) were collected onto SuperFrost^®^plus glass slides (Langenbrinck) and stored at −70 °C until immunohistochemical labeling was performed as described for whole-mount preparations. For negative control the ED and ES epithelium of rat p4 and ES epithelium of rat p31 were stained with the same protocol without primary antibodies (Supplementary Fig. S7). Microscopic analysis was performed with a Zeiss ApoTome.2 (Zeiss, Oberkochen, Germany) or a Zeiss 510 Meta confocal laser scanning microscope (Zeiss).

### Pharmacological AVP stimulation of the ED and ES

To test the regulatory effect of AVP on tight –junction proteins in the ED and ES epithelium, the V2R specific agonist 1-Desamino-8-D-Arginin-Vasopressin (dDAVP; Bachem, Bubendorf, Switzerland) in the range of physiological concentrations of 10^−6^ M, 10^−8^ M and 10^−10^ M and of the V2R antagonist H-9400 (concentrations: 10^−6^ M μM, 10^−8^ M and 10^−10^ M; Bachem, Bubendorf, Switzerland) were added to the DMEM/F12 medium without HEPES supplemented with 25% horse serum, 10 IU/ml penicillin/streptomycin, 2 mM L-glutamine, 0,9 mg/ml sodium bicarbonate (ThermoFisher Scientific)^114^, respectively. Incubation of ES-specimens was performed for 30 min, 1 h, and 3 h. The range of physiological concentration values^115^ and time periods were selected from former studies dealing with AVP stimulation experiments in the ES and the kidney^100^,^116^,^117^. Specimens were then fixed and processed for immunohistology as described above.

## Additional Information

**How to cite this article**: Runggaldier, D. *et al*. Claudin expression in the rat endolymphatic duct and sac - first insights into regulation of the paracellular barrier by vasopressin. *Sci. Rep.* **7**, 45482; doi: 10.1038/srep45482 (2017).

**Publisher's note:** Springer Nature remains neutral with regard to jurisdictional claims in published maps and institutional affiliations.

## Supplementary Material

Supplementary Information

## Figures and Tables

**Table 1:** Assesment of absolute Claudin mRNA expression in rat p4 ED, ES and ST.

Gene	ED	ES	ST	Gene	ED	ES	ST	Gene	ED	ES	ST
Cldn1	+	++	+	Cldn9	+	+	(+)	Cldn18	n.d.	n.d.	n.d.
Cldn3	(+)	(+)	(+)	**Cldn10**	**++++**	**+++++**	**++**	Cldn19	(+)	(+)	++
**Cldn3**	**+++**	**++++**	**+**	Cldn11	++++	+++	++++	Cldn20	++	++	++
**Cldn4**	**+++**	**++++**	**+**	Cldn12	+	++	+	Cldn22	+	+	++
Cldn5	+	+	++	Cldn14	n.d.	n.d.	n.d.	Cldn23	+	+	+
**Cldn6**	**+++**	**++++**	**+**	Cldn15	+	+	+	Cldn24	+	+	+
**Cldn7**	**++**	**+++**	**+**	**Cldn16**	**++**	**+++**	**+**	Pend	+++	+++++	++
**Cldn8**	**+++++**	**+++++**	**++**	Cldn17	++	++	(+)	UBC	++++	++++	++++

Ct ≤ 11: +++++ very high expression; 14 < Ct ≤ 17: +++ moderate expression; 17 < Ct ≤ 20: ++ low expression; 20 < Ct ≤ 24: ++ very low expression; Ct > 24: (+) extremely low expression; n.d.: not detected.

**Table 2:** TaqMan Gene Expression Assays (Thermo Fisher Scientific).

Gene Symbol	Assay ID	Gene Symbol	Assay ID	Gene Symbol	Assay ID
Claudin 1 (Cldn1)	Rn00581740_m1	Claudin 9 (Cldn9)	Rn01460292_s1	Claudin 18 (Cldn18)	Rn01447445_m1
Claudin 2 (Cldn2)	Rn02063575_s1	Claudin 10 (Cldn10)	Rn01468224_m1	Claudin 19 (Cldn19)	Rn01416537_m1
Claudin 3 (Cldn3)	Rn00581751_s1	Claudin 11 (Cldn11)	Rn00584941_m1	Claudin 20 (Cldn20)	Rn01430561_s1
Claudin 4 (Cldn4)	Rn01196224_s1	Claudin 12 (Cldn12)	Rn04219013_m1	Claudin 22 (Cldn22)	Rn03416360_s1
Claudin 5 (Cldn5)	Rn01753146_s1	Claudin 14 (Cldn14)	Rn01407193_m1	Claudin 23 (Cldn23)	Rn01482199_s1
Claudin 6 (Cldn6)	Rn01464110_m1	Claudin 15 (Cldn15)	Rn04244372_m1	Claudin 24 (Cldn24)	Rn01487934_s1
Claudin 7 (Cldn7)	Rn01496517_g1	Claudin 16 (Cldn16)	Rn00590884_m1		
Claudin 8 (Cldn8)	Rn01496517_g1	Claudin 17 (Cldn17)	Rn01771991_s1		
**Control and Reference Genes**					
Pendrin (Scl26a4)	Rn01469208_m1	Tata Box Binding (TBP)	Rn01455646_m1	Ubiquitin C (UBC)	Rn01789812_g1

**Table 3:** Antibodies.

Primary Antibodies	Secondary Antibodies
rabbit anti-Cdl3, 1:100, Thermo Fisher Scientific	Donkey anti-rabbit Alexa 546, 1:400, Thermo Fisher Scientific
rabbit anti-Cld4, 1:100, Thermo Fisher Scienttific	Donkey anti-rabbit Alexa 546, 1:400, Thermo Fisher Scientific
rabbit anti-Cld6, 1:100, IBL International	Donkey anti-rabbit Alexa 546, 1:400, Thermo Fisher Scientific
rabbit anti-Cld7, 1:100, IBL International	Donkey anti-rabbit Alexa 546, 1:400, Thermo Fisher Scientific
rabbit anti-Cld8, 1:100, Thermo Fisher Scientific	Donkey anti-rabbit Alexa 546, 1:400, Thermo Fisher Scientific
rabbit anti-Cld8, 1:100, IBL International	Donkey anti-rabbit Alexa 546, 1:400, Thermo Fisher Scientific
rabbit anti-Cld10, 1:100, Thermo Fisher Scientific	Donkey anti-rabbit Alexa 546, 1:400, Thermo Fisher Scientific
rabbit anti-Cld16, 1:100, Thermo Fisher Scientific	Donkey anti-rabbit Alexa 546, 1:400, Thermo Fisher Scientific
mouse anti-ZO1, 1:100, Thermo Fisher Scientific	Donkey anti-mouse Alexa 488, 1:400, Thermo Fisher Scientific
