# Supplementary material for: Claudin expression in the rat endolymphatic duct and sac - first insights into regulation of the paracellular barrier by vasopressin
Source: Sci Rep. 2017 Apr 4;7:45482. doi: 10.1038/srep45482 (PMC5379655; doi:10.1038/srep45482)
Supplement: Supplementary Information [file srep45482-s1.pdf]

Supplementary information

Claudin expression in the rat endolymphatic duct and sac - first insights into regulation of the paracellular barrier by vasopressin

Authors: Daniel Runggaldier, Lidia Garcia Pradas, Peter Neckel, Andreas Mack, Bernhard Hirt, Corinna Gleiser

The following pages include:

Supplementary Figure S1
Supplementary Table S1
Supplementary Figure S2
Supplementary Figure S3
Supplementary Figure S4
Supplementary Figure S5
Supplementary Figure S6
Supplementary Figure S7
Supplementary Figure S8

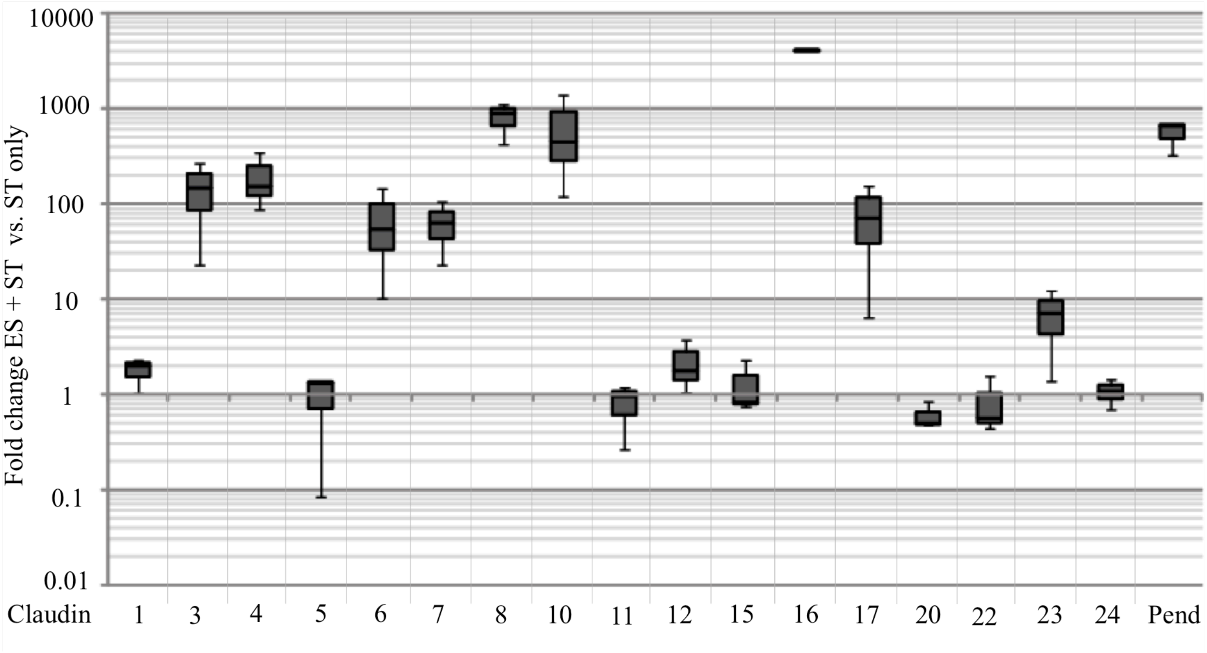

Supplementary Figure S1: Claudin mRNA expression in rat p31 ES epithelium High throughput qPCR analysis of p31 ES plus tightly attached surrounding tissue (ST) mRNA versus surrounding tissue mRNA. TaqMan probes against exon-exon boundaries of all claudin mRNA subtypes were used (Table 1). Box-whisker-blots show 25 – 75 percentile of the normalized relative quantities (NRQs) of rat p31 ES (black bars) plus attached surrounding tissue claudin mRNA expression versus claudin mRNA expression of surrounding tissue. Claudin 3, 4, 6, 7, 8, 10, 16, 17 and 23 mRNA were found to be expressed considerable stronger in the ES with a NRQ of 10 or more if compared with sourrounding tissue. Claudins not shown were not expressed in the ES or ED (n = 3). Positive control target: Pendrin (Pen).

Supplementary Table S1: Assessment of absolute Claudin mRNA expression in rat p31 ES plus surrounding tissue and surrounding tissue

Gene	P30 ES + ST	ST	Gene	P30 ES + ST	ST	Gene	P30 ES + ST	ST
Cldn1	++	++	Cldn9	++	+	Cldn18	n.d.	n.d.
Cldn2	(+)	(+)	**Cldn10**	**+++++**	**++**	Cldn19	(+)	(+)
**Cldn3**	**++++**	**++**	Cldn11	++++	+++<br>+	Cldn20	+++	+++
**Cldn4**	**+++++**	**++**	Cldn12	++	++	Cldn22	+++	+++
Cldn5	++	++	Cldn14	n.d.	n.d.	Cldn23	+	+
**Cldn6**	**+++++**	**++**	Cldn15	++	++	Cldn24	++	++
**Cldn7**	**+++**	**+**	**Cldn16**	**+++**	(+)	Pend	+++++	++
**Cldn8**	**+++++**	**++**	Cldn17	++	+	UBC	+++++	+++++

$Ct \leq 11$: +++++ very high expression; $14 < Ct \leq 17$: +++ moderate expression; $17 < Ct \leq 20$: ++ low expression; $20 < Ct \leq 24$: ++ very low expression; $Ct > 24$: (+) extremely low expression; n.d.: not detected

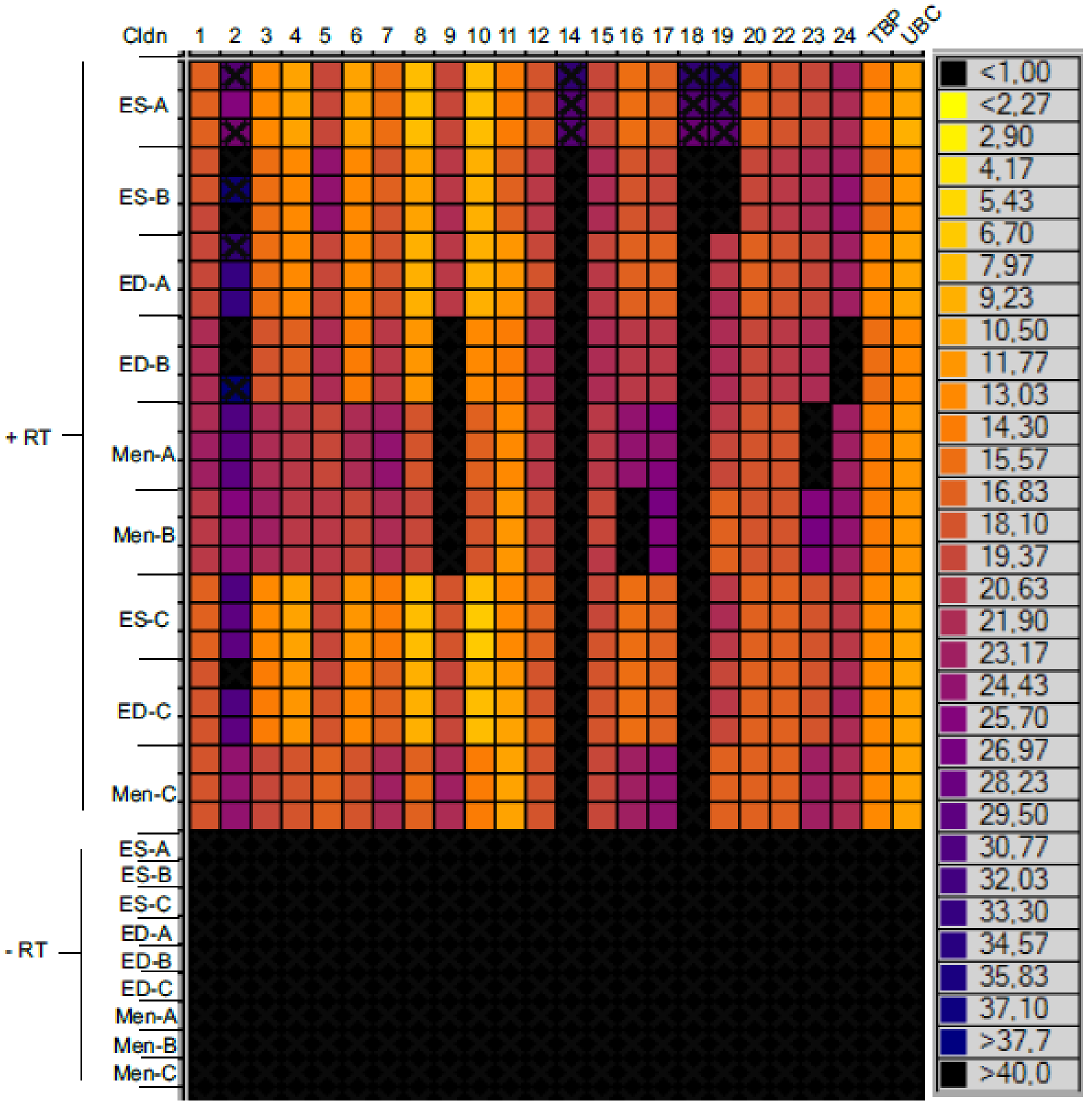

Supplementary Figure S2: Results from one experiment on Fluidigm's Dynamic Array presented as a heat map with individual assays on the *x* axis and individual tissue samples on the *y* axis. The intersection of each assay and sample is an individual real-time quantitative PCR result.The colors on the heat map correspond to the Ct values, as indicated in the legend. The heat map legend shows color-coded Ct values for every reaction position on the dynamic array. For negative controls, the Reverse Transcriptase was replaced with nuclease-free water (- RT).

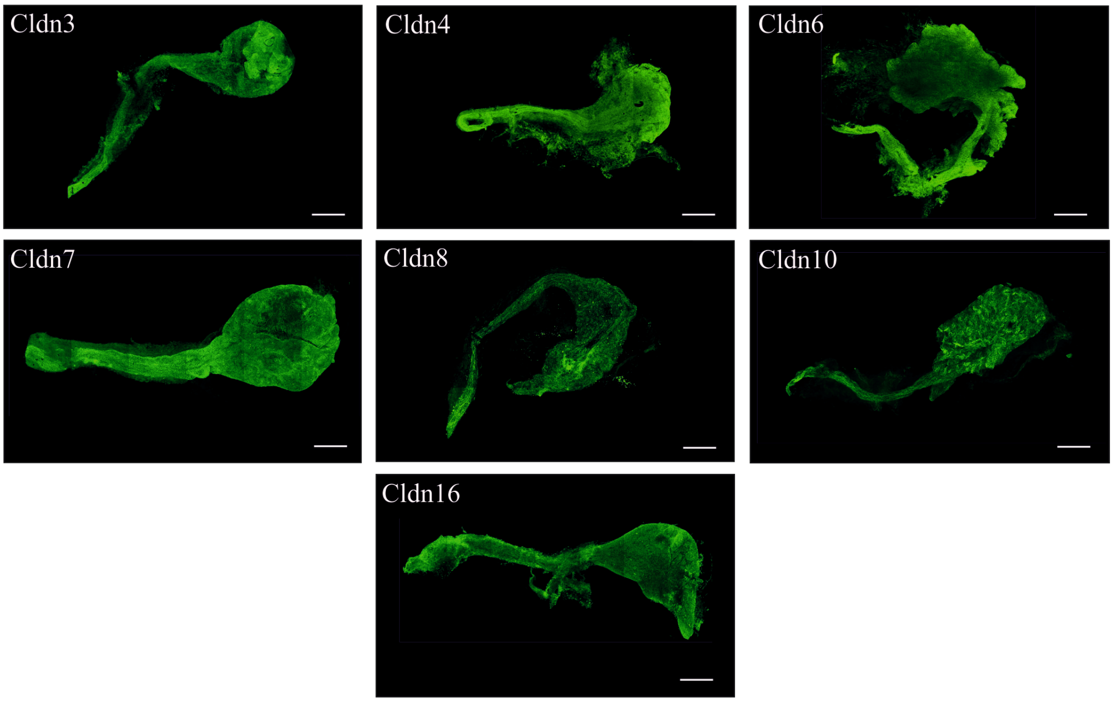

Supplementary Figure S3: Expression of claudin proteins in the ES and adhering ED. Mosaic images of optical sections covering the complete ES and adhering ED specimens (rat p4) stained with the indicated claudin subtype antibodies followed by Alexa488-conjuncated anti-rabbit antiserum acquired with structured illumination microscopy (apotome). Scale bars: 10 µm.

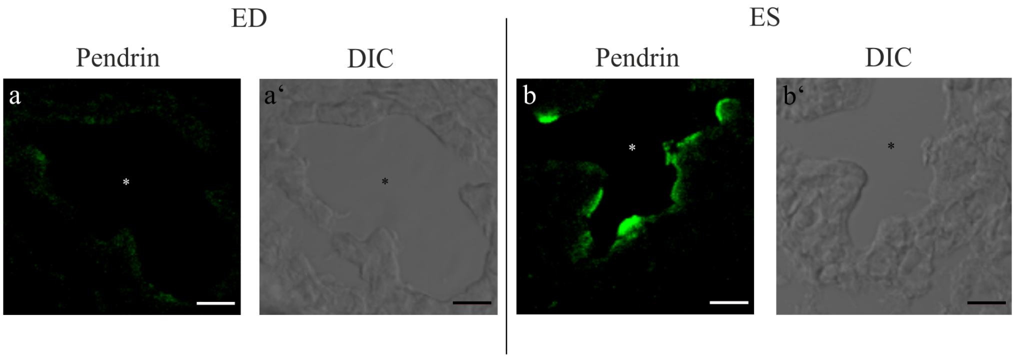

Supplementary Figure S4: cLSM analysis of the subcellular immunolocalization of pendrin in the rat p4 ED and ES epithelium. (a) cLSM image of Pendrin labelling and (a') the corresponding differential interference contrast (DIC) image in a section of the rat p4 ED. Almost no pendrin immunolabeling consistent with the low distribution of mitochondria-rich cells in the ED epithelium was observed. (b) cLSM image of Pendrin labeling and (b') the corresponding DIC image. In the mitochondria-rich cells of the ES epithelium pendrin immunolabelling was observed in the apical membrane domains. Scale bars: 10 µm.

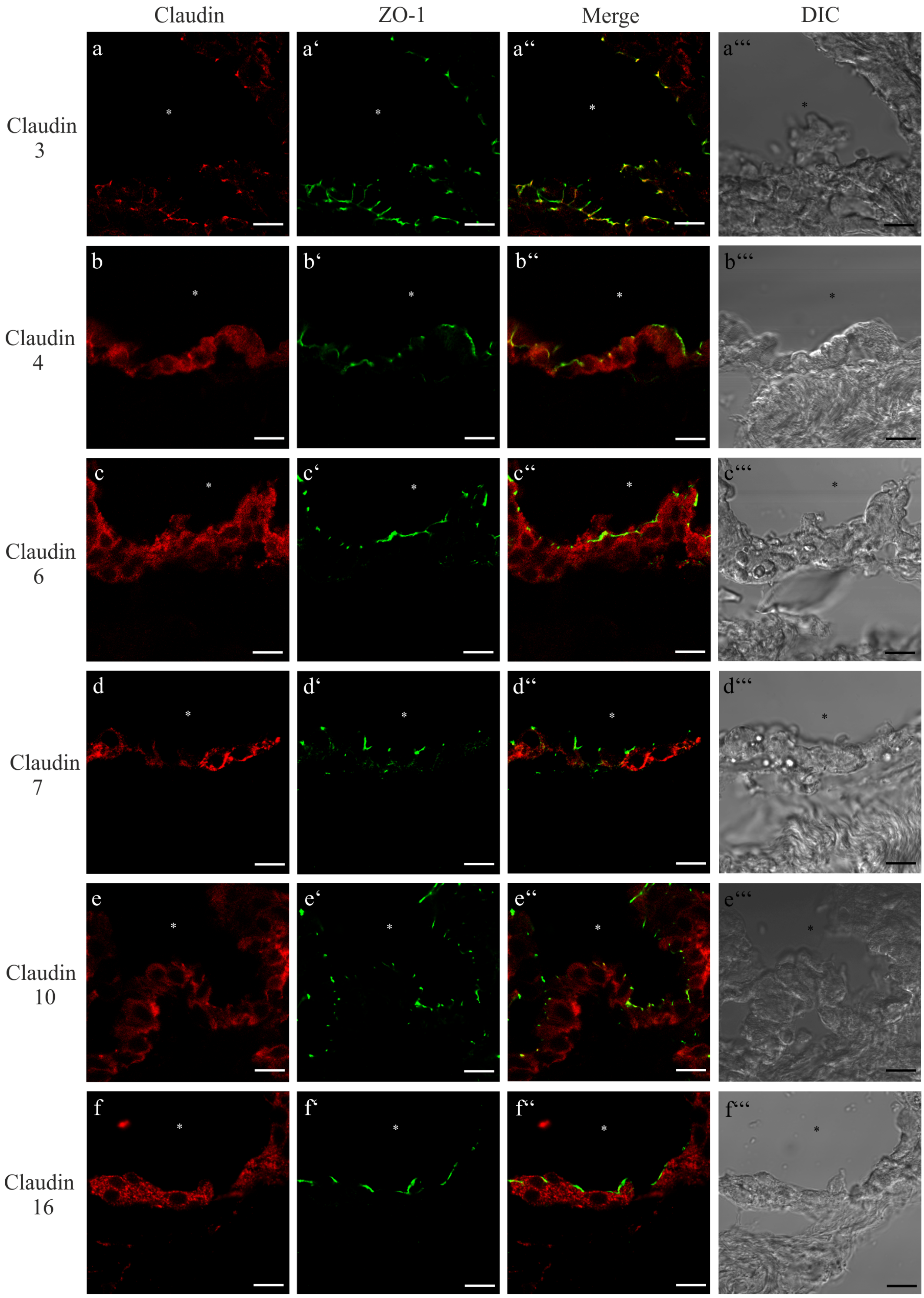

Supplementary Figure S5: cLSM analysis of the subcellular immunolocalization of Claudin 3, 4, 6, 7, 10 and 16 in the rat p31 ES epithelium. Cryosections of rat p31 ES plus attached surrounding tissue were co-localized with the indicated claudin subtype antibodies and ZO1 antibody. (a, b, c, d, e, f) cLSM image of the respective claudin proteins labelling in adult ES epithelium. (a’, b’, c’, d’, e’, f’) cLSM image of

ZO-1 labelling in the corresponding cryosection of.adult ES epithelium. (a'', b'',c'', d'', e'', f'') Merged images of the respective Claudin proteins and ZO-1 immunolabelling in the adult ES epithelium. (a''', b''', c''', d''', e''', f''') Corresponding differential interference contrast (DIC) image of the ES epithelium. (a – a''') Claudin 3 in the ES epithelium is co-localized with ZO1 in the TJs. (b – b''') Cytoplasmic Claudin 4 expression was observed in the ES epithelial cells. (c-c''') Claudin 6 immunolabelling was detected in the cytoplasm of the ES epithelial cells. (d – d''') Claudin 7 immunolabelling was localized in the basolateral membranes of the ES epithelial cells. (e – e''') The epithelial cells of the ES exhibited a apical and cytoplasmic Claudin 10 immunolabelling. (f – f''') Claudin 16 immunolabelling was found in the cytoplasm of the ES epithelial cells. Scale bars: 10 µm.

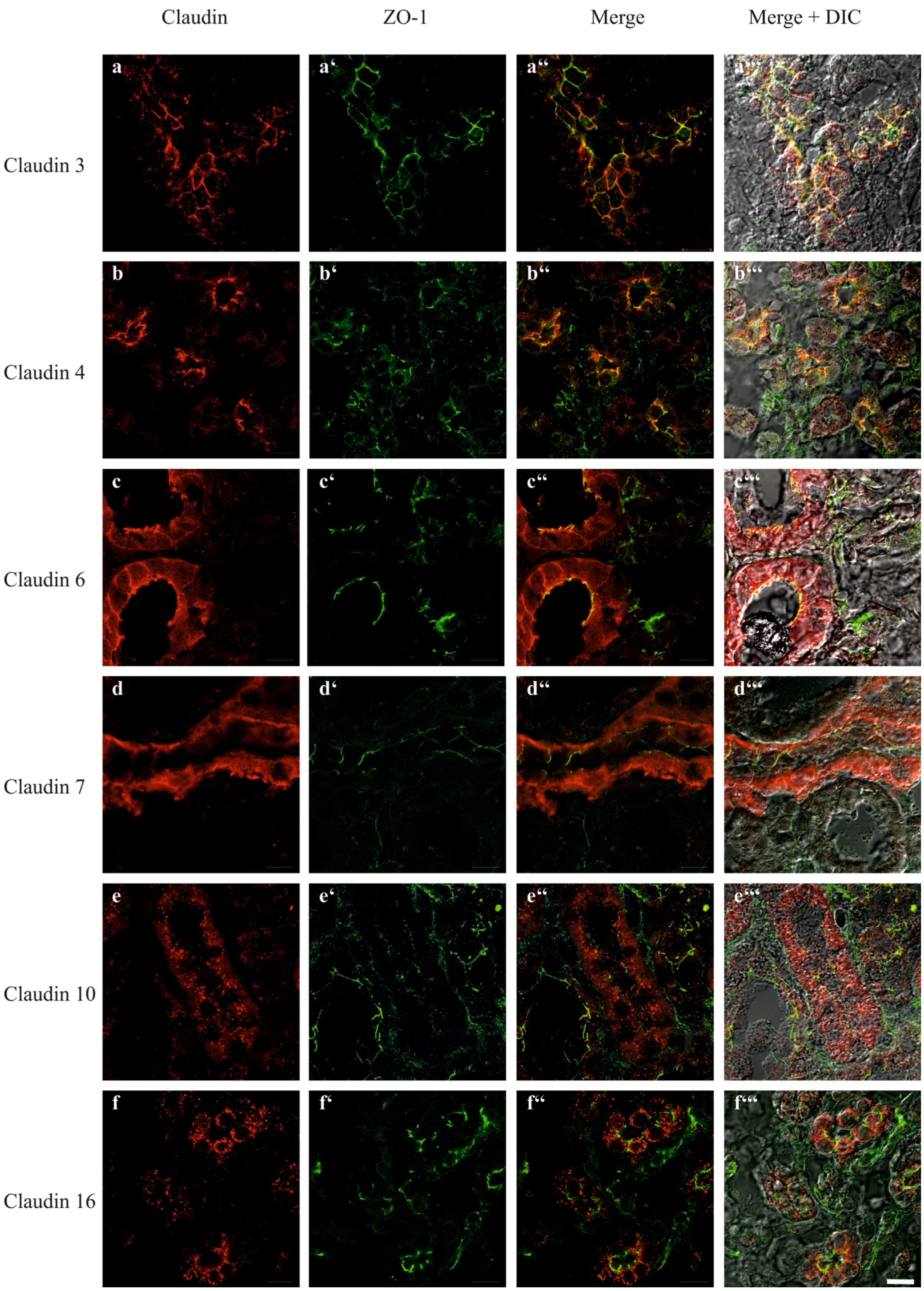
Claudin
ZO-1
Merge
Merge + DIC
Claudin 3
Claudin 4
Claudin 6
Claudin 7
Claudin 10
Claudin 16
a
a‘
a‘‘
a‘‘‘
b
b‘
b‘‘
b‘‘‘
c
c‘
c‘‘
c‘‘‘
d
d‘
d‘‘
d‘‘‘
e
e‘
e‘‘
e‘‘‘
f
f‘
f‘‘
f‘‘‘

Supplementary Figure S6: cLSM analysis of the subcellular immunolocalization of Claudin 3, 4, 6, 7, 10 and 16 in the rat p3 kidney. Cryosections of rat p3 kidney were co-localized with the indicated claudin subtype antibodies and ZO1 antibody. (a, b, c, d, e, f) cLSM image of the respective Claudin proteins labelling in kidney. (a', b', c', d', e', f') cLSM image of ZO-1 labelling in the corresponding cryosection of.kidney. (a'', b'',c'', d'', e'', f'') Merged images of the respective Claudin proteins and ZO-1 immunolabelling in the kidney. (a''', b''', c''', d''', e''', f''') Corresponding differential interference contrast (DIC) image of the kidney cryosection. (a-a''') Claudin 3 is co-localized with ZO1 in the TJs of the thick ascending limbs of Henle. (b-b''') Claudin 4 expression was observed in the TJs of the thin ascending limbs of Henle. (c-c''') Claudin 6 immunolabelling was detected in the cytoplasm and basolateral membrane domains of the epithelial cells of the collecting duct. (d – d''') Claudin 7 immunolabelling was localized in the basolateral membranes of the collecting duct epithelial cells. (e – e''') The epithelial cells of the distal tuble exhibited a cytoplasmic Claudin 10 immunolabelling. (f – f'') Claudin 16 immunolabelling was found in the basolateral membranes of the thick ascending limbs. Scale bar: 10 µm.

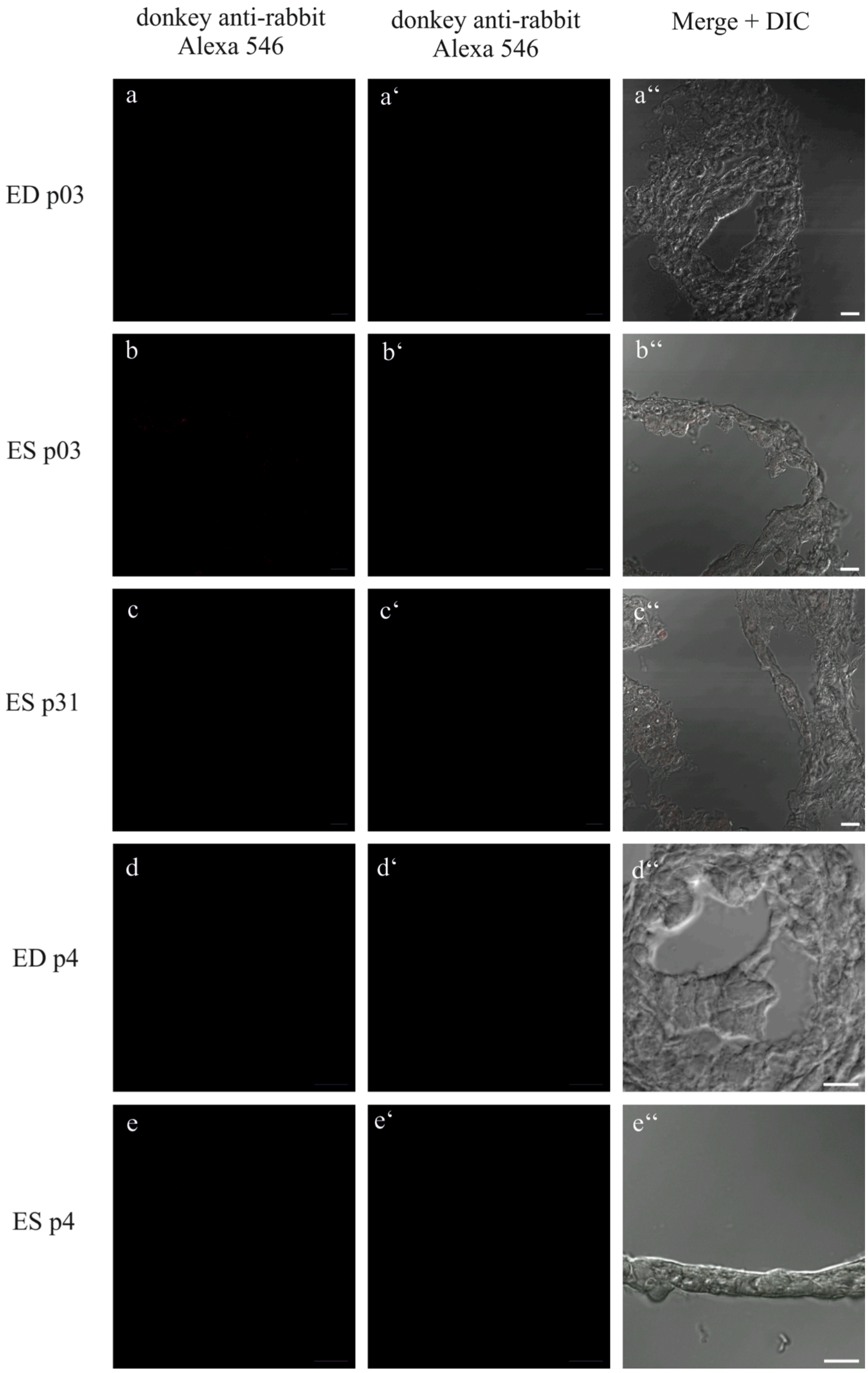

Supplementary Figure S7: Control for nonspecific binding of the secondary antibodies. For negative control the ED and ES epithelium of rat p4 and ES epithelium of rat p31 were stained with the same protocol as the other immunohistochemical analyses without primary antibodies. (a, b, c, d, e) cLSM image of the donkey anti-rabbit Alexa 546 staining in ED and ES epithelium. (a’, b’, c’, d’, e’) cLSM image of donkey anti-rabbit Alexa 488 staining in the corresponding

cryosection of ED and ES epithelium. (a'', b', c'', d'', e'', f'') Merged images of the stained secondary antibodies and the corresponding differential interference contrast (DIC) image of the ED and ES epithelium. (d-e'') The stained (without primary antibodies) ED and ES epithelium of rat p4 was analyzed by confocal microscopy with the same settings used for the other microscopic analyses. In the negative controls no specific secondary antibody binding was detected.

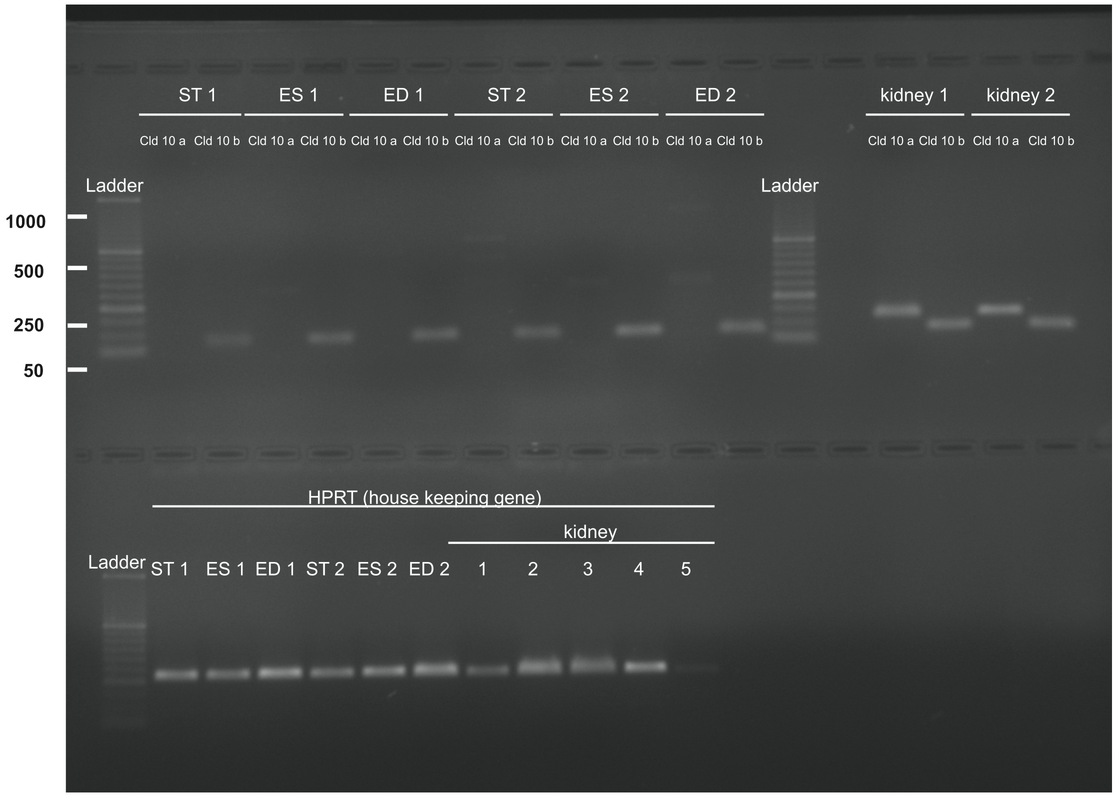

Supplementary Figure S8: Original image of cropped gels in Figure 3 b
